# Supplementary material for: Comprehensive Multi-Omic Evaluation of the Microbiota and Metabolites in the Colons of Diverse Swine Breeds
Source: Animals (Basel). 2024 Apr 18;14(8):1221. doi: 10.3390/ani14081221 (PMC11047667; doi:10.3390/ani14081221)
Supplement: Supplementary file 1 [file animals-14-01221-s001.zip › Supplementary Figure S1.pdf]

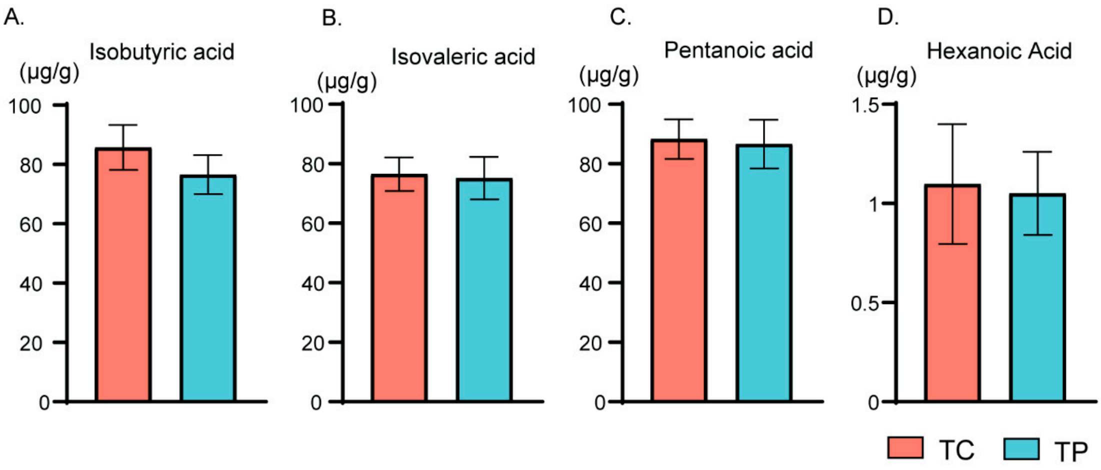

Supplementary Figure S1. **Different concentrations of SCFAs in colon of two breeds pigs.** (A) Isobutyric acid (ug / g), (B) Isovaleric acid (ug / g), (C) Pentanoic acid (ug / g), and (D) Hexanoic Acid (ug / g) in the colonic samples from TC and TP pigs. Data are presented as mean ± SD and statistical significance was determined by the Wilcoxon rank-sum test; TC, Duroc × landrace × yorkshire pigs; TP, Tibetan pig; *represents significantly difference ($P \leq 0.05$), **$P \leq 0.01$ and ***$P \leq 0.001$; n = 6.
