# Supplementary material for: Comprehensive Multi-Omic Evaluation of the Microbiota and Metabolites in the Colons of Diverse Swine Breeds
Source: Animals (Basel). 2024 Apr 18;14(8):1221. doi: 10.3390/ani14081221 (PMC11047667; doi:10.3390/ani14081221)
Supplement: Supplementary file 1 [file animals-14-01221-s001.zip › Supplementary Table S1.pdf]

Table S1. Primers used for real-time quantitative PCR analysis

Genes	Primers	Primers sequences (5’ to 3’)
FXR	F	TATGAACTCAGGCGAATGCCTGCT
	R	ATCCAGATGCTCTGTCTCCGCAAA
TGR5	F	CCATGCACCCCTGTTGCT
	R	GGTGCTGTTGGGTGTCATCTT
GPR41	F	GCCCTTGCCCTTCATCTTCT
	R	CCGGGTCTTGTACCAGAGTG
GPR43	F	CAGAGGCAAAGAGACCGAGG
	R	TGGTGAAGTCAGAACTCGGC
GPR109	F	CGCGATTTCCCAGAACTTCC
	R	AGCTTCAGACGCCTAGGAAC
SLC5A8	F	GGCACTCGTTTGTGAAGCTG
	R	ATCCGCCCTCCCAAACATTC
SLC16A1	F	CATCAACTACCGACTTCTG
	R	TACTGGTCTCCTCCTCTT
β-actin	F	TCTGGCACCACACCTTCT
	R	TGATCTGGGTCATCTTCTCAC
