# Supplementary material for: Comprehensive Multi-Omic Evaluation of the Microbiota and Metabolites in the Colons of Diverse Swine Breeds
Source: Animals (Basel). 2024 Apr 18;14(8):1221. doi: 10.3390/ani14081221 (PMC11047667; doi:10.3390/ani14081221)
Supplement: Supplementary file 1 [file animals-14-01221-s001.zip › Supplementary Table S2.pdf]

Supplementary Table S2. Identified metabolites in colon between Three-way crossbred pigs and Tibetan pigs based on the untargeted metabolomics study.

Metabolite name	VIP	P-value	Mean-Tibetan	Mean-Three-way crossbred
3-hydroxyphenylacetic acid	1.23408367	0.00994071	0.00493418	0.00852868
Tranexamic acid	1.61583781	0.04016464	0.01241007	0.03897431
Ornithine	1.02844892	0.01115690	0.03341698	0.05195989
Maltotriose	2.00903675	0.01011463	0.01654747	0.05888891
D-tagatose	1.74151184	0.00370724	0.00887009	0.02804889
Methyl beta-d-glucopyranoside	1.72126727	0.01330937	0.00756801	0.02316986
Phytol	1.76523843	0.04501250	0.00008520	0.00031054
Tryptophol	1.67730697	0.00791388	0.00027518	0.00083882
2'-deoxyguanosine	1.21867737	0.01070947	0.00277620	0.00475074
3-fluoro-2-hydroxyprop-2-enoate	1.38269551	0.00950902	0.00015439	0.00030115
2,5-di-tert-butyl-4-(hydroxy)phenol	1.32199431	0.00081788	0.00086014	0.00048539
Scyllo-inositol	2.11798817	0.00891241	0.04843655	0.01185180
2-hydroxybutanoic acid	1.62675891	0.01559441	0.00287352	0.00087185
L-lactic acid	2.08047308	0.00022205	0.49133808	0.14094746
Pyruvic acid	1.57943087	0.01183818	0.01005537	0.00466033
4-aminobutyric acid	1.23761591	0.01660566	0.01733129	0.00938658
4-hydroxyproline	1.45797278	0.02035116	0.00161683	0.00065715
Alpha-aminoadipic acid	1.67493819	0.04067954	0.00104401	0.00025778
Aminomalonate	1.92286601	0.00469622	0.00215117	0.00060636
Dl-dopa	1.04687476	0.02013519	0.06265073	0.04143469
L-aspartic acid	1.38347192	0.00415603	0.15280830	0.07800620
L-cysteine	1.63936771	0.00004206	0.00232711	0.00100919
L-methionine	1.23816849	0.01270627	0.01631765	0.00928174
L-phenylalanine	1.08602672	0.02043931	0.02690502	0.01720934
L-threonine	1.15390470	0.00362977	0.02364921	0.01480341
Sarcosine	1.26195079	0.03885478	0.02971730	0.01597884
Serine	1.18558256	0.00518708	0.06360789	0.03861694
Indoxyl sulfate	1.09954947	0.01682029	0.01554826	0.00891564
Phthalic acid	2.60552252	0.00034143	0.00062146	0.00007404
Syringic acid	1.19430854	0.04307017	0.00192721	0.00111042
Hydroxypropionic acid	2.06630460	0.00179578	0.03168541	0.00785386

Lithocholic acid	1.76272827	0.04200230	0.03556632	0.01283067
Biphenyl	1.49733797	0.00098841	0.00523124	0.00245740
Digalacturonic acid	1.41789964	0.00187148	0.00290796	0.00151483
Erythronic acid	1.55472451	0.00942964	0.00031062	0.00013535
Gluconic acid	2.23868396	0.00937416	0.01303349	0.00211217
Cholesterol	1.06351809	0.02901781	0.00382156	0.00229530
2-hydroxy-2-methylbutanoic acid	1.18629601	0.01496885	0.00566068	0.00303304
2-isopropylmalic acid	1.28940417	0.00027835	0.00033167	0.00018973
3-methyladipic acid	1.88711224	0.00016203	0.00488476	0.00161858
Citramalic acid	1.58144291	0.03165688	0.01451394	0.00708545
Elaidic acid	2.42877894	0.00225380	0.28957917	0.05635958
Heptadecanoic acid	1.95242704	0.03263250	0.06776604	0.02467997
Hexadecanedioic acid	1.17405291	0.01328814	0.00115516	0.00063649
Nonadecanoic acid	1.35274621	0.03195327	0.00150317	0.00080257
Oleic acid	2.41596727	0.00225418	0.28931339	0.05667671
Palmitic acid	1.62988583	0.00864833	1.18020020	0.52106756
Pentacosanoic acid	2.27463565	0.00451420	0.31369632	0.09010349
Sebacic acid	2.06925899	0.00018969	0.25678174	0.06427830
Tridecanoic acid	2.37087185	0.00043531	0.02230124	0.00459101
Dehydroascorbic acid	1.18986622	0.00703886	0.06980261	0.04139313
Urocanic acid	1.02735012	0.01598114	0.00359300	0.00239468
Linoleic acid	2.05013983	0.00477167	0.02652067	0.00804687
Taurine	2.12725613	0.01428221	0.00080077	0.00019007
Orotic acid	1.22399646	0.01939245	0.00202152	0.00112316
Kynurenic acid	1.40697746	0.00273551	0.00361348	0.00173345
L-2-hydroxyglutaric acid	1.65738290	0.03861829	0.01436165	0.00592735
2-ketobutyric acid	1.36710228	0.02787210	0.00213142	0.00113347
Isocitric acid	1.17127020	0.00934335	0.06874154	0.04120830
2-hydroxy-2-phenylpropanoic acid	1.17763793	0.02050273	0.00048541	0.00026452
3-methylene-2-phenyl-1-hydroxy-cyclopentene	1.83588681	0.00550959	0.00053459	0.00015065
4-hydroxyanthraquinone-2-carboxylic acid	1.45106049	0.03203373	0.02426230	0.01303426
6-deoxyglucitol	1.12311052	0.00067842	0.00062894	0.00040828
Alloxanoic acid	1.26175283	0.01846038	0.00019561	0.00009874
Digitoxose	1.11556611	0.00278112	0.01659349	0.01078277
Dihydroxymalonic acid	1.51214209	0.00085561	0.00198514	0.00086095

Harmol	1.37879717	0.00520894	0.00018092	0.00009696
N-(2-hydroxyethyl) tetradecan-1-amine	1.29189540	0.00121909	0.00462236	0.00262804
Phosphenodiimidic amide	1.50620834	0.00020567	0.00096904	0.00047872
