# Supplementary material for: Comprehensive Multi-Omic Evaluation of the Microbiota and Metabolites in the Colons of Diverse Swine Breeds
Source: Animals (Basel). 2024 Apr 18;14(8):1221. doi: 10.3390/ani14081221 (PMC11047667; doi:10.3390/ani14081221)
Supplement: Supplementary file 1 [file animals-14-01221-s001.zip › Supplementary Table S3.pdf]

Supplementary Table S3. Identified metabolites in plasma between Three-way crossbred pigs and Tibetan pigs based on the untargeted metabolomics study.

**Metabolite name**	**VIP**	**P-value**	**Mean-Tibetan**	**Mean-Three-way crossbred**
Quinic acid	1.33935046	0.01905904	0.00049732	0.00022845
Bicine	1.78928405	0.00081585	0.00481059	0.00201228
L-histidine	1.46853866	0.00524562	0.07049220	0.03759828
Homocystine	1.44541666	0.03440558	0.00027871	0.00010160
L-cystine	1.35266807	0.01879328	0.00586259	0.00309301
L-asparagine	1.15854946	0.00843793	0.00762664	0.00488002
Malic acid	1.74660289	0.00061887	0.08619464	0.03730567
D-tagatose	1.93588309	0.00144981	0.04175187	0.01533489
D-arabinose	1.79200480	0.00004090	0.00549308	0.00237999
Maltotriose	1.58704381	0.02554653	0.02012467	0.00632141
Coniferin	1.57741699	0.02088390	0.00398960	0.00136344
L-sorbose	1.55800045	0.02235854	0.02240736	0.00971204
1,5-anhydroglucitol	1.42058454	0.02392643	0.03545460	0.01796404
D-fructose	1.23235629	0.02640481	0.01164541	0.00664570
N-acetylputrescine	1.64592557	0.00067227	0.02567969	0.01241198
Cholesterone	1.26911482	0.00160302	0.00090025	0.00056944
Fumaric acid	1.56159323	0.00037042	0.02889687	0.01482558
Glutaric acid	1.27859150	0.00385307	0.00095121	0.00058398
Acifluorfen	1.51012147	0.01601210	0.01063043	0.00460075
Hexadecanedioic acid	1.60195252	0.02686166	0.00239884	0.00086885
Tetradecanedioic acid	1.34274717	0.03159409	0.00311370	0.00157152
Lactitol	2.11090530	0.00060722	0.00049127	0.00015643
5-methoxyindoleacetate	1.83675223	0.00071689	0.04875821	0.01941305
Linoleic acid	1.44735755	0.01380474	0.01500739	0.00790926
Metanephrine	1.55842614	0.00004206	0.00303996	0.00161591
Taurine	1.42717422	0.00431699	0.00683037	0.00376021
O-phosphoethanolamine	1.41492840	0.01074643	0.02461763	0.01258863
2'-deoxyguanosine	1.69102629	0.00447574	0.01164028	0.00472661
Hypoxanthine	1.45440987	0.00061117	0.34001102	0.18650466
Gamma-tocopherol	2.45090806	0.00063430	0.00170382	0.00036512
Beta-sitosterol	1.49474582	0.01082124	0.00082741	0.00036776
4-methyl-5-thiazoleethanol	2.58926363	0.00877082	0.08049209	0.01101100
3,17,20-trihydroxy-pregn-5-en-11-one	2.75437082	0.00072479	0.02022636	0.00297648
Inosine	2.48533559	0.00145987	0.05383478	0.00917989

6-hydroxy-alpha-methylnaphthaleneacetic acid	1.75815471	0.00049659	0.37068385	0.15890593
6-hydroxy-2-aminohexanoic acid	1.43026060	0.04703146	0.08383422	0.03472409
Alloxanic acid	1.18476553	0.00955663	0.01375182	0.00867045
Palatinitol	1.02620892	0.01515769	0.01772263	0.01143086
2-aminoheptanedioic acid	2.50396135	0.02850517	0.00157164	0.01477819
N-carbamoylaspartate	1.76907659	0.02920518	0.00046267	0.00172310
Aminomalonate	1.67714985	0.00900832	0.05977971	0.14923040
Creatinine	1.36400509	0.00619386	0.04411832	0.08258905
Pipecolic acid	1.29833642	0.00358589	0.00195650	0.00319867
Mesalazine	2.29966016	0.02716268	0.00079912	0.00559820
Glucosamine	2.27594497	0.00022667	0.00004879	0.00019458
D-fructose-1-phosphate	1.60136486	0.00373796	0.00345932	0.00777640
Glucose-1-phosphate	1.28043617	0.00039241	0.00885973	0.01396275
Arabinofuranose	1.20660713	0.00481612	0.00383280	0.00598486
Glucose	1.15265472	0.02939493	0.38926904	0.62623756
3-phosphoglyceric acid	1.08630755	0.03164072	0.00563794	0.00887462
P-cresol	2.08893982	0.00045214	0.00125938	0.00395927
Beta-hydroxymyristic acid	2.85267465	0.00370812	0.00015336	0.00182296
Lauric acid	2.70414137	0.01632372	0.00028513	0.00298727
Dodecanol	1.40733479	0.00008670	0.00212087	0.00365123
Dehydroascorbic acid	2.30035492	0.01561389	0.00024649	0.00187631
Adenine	1.40199902	0.00412212	0.00648310	0.01152360
N-acetyl-d-tryptophan	2.26673385	0.01275369	0.00158827	0.00936890
13beta-ethyl-3-oximinogon-4-en-17-one	2.09952307	0.02463103	0.00099066	0.00567703
Lactobionic acid	2.06525006	0.00788088	0.00050376	0.00191631
4-(dimethylamino)azobenzene	2.00090369	0.01275786	0.00038203	0.00147625
Glycyl tyrosine	1.84519555	0.01103718	0.00800688	0.02754555
Dihydroxymalonic acid	1.83575290	0.00297359	0.00035339	0.00096695
Phosphenodiimidic amide	1.81288506	0.00157431	0.00374384	0.00966586
2,3-dihydro-8-methoxyfuro(2,3-b)quinoline	1.61856063	0.01490803	0.00128337	0.00321977
Piceatannol	1.56036045	0.04843402	0.00535176	0.01882930
Xanthosine	1.41708946	0.00040427	0.00595154	0.01056584
L-cysteine-glycine	1.41707709	0.00032199	0.00044009	0.00078030
L-glutamine dehydrated	1.14792517	0.00887382	0.00610824	0.00965092
